# Alpha-Lipoic Acid Promotes Intestinal Epithelial Injury Repair by Regulating MAPK Signaling Pathways

**DOI:** 10.1155/2022/1894379

**Published:** 2022-06-07

**Authors:** Yu Yang, Yong Xiao, Yue Jiang, Jiajun Luo, Jingwen Yuan, Junfeng Yan, Qiang Tong

**Affiliations:** ^1^Department of Gastrointestinal Surgery I Section, Renmin Hospital of Wuhan University, Wuhan 430060, China; ^2^Department of Gastroenterology, Renmin Hospital of Wuhan University, Wuhan 430060, China

## Abstract

Intestinal epithelial cells are an essential barrier in human gastrointestinal tract, and healing of epithelial wound is a key process in many intestinal diseases. *α*-Lipoic acid (ALA) was shown to have antioxidative and anti-inflammatory effects, which could be helpful in intestinal epithelial injury repair. The effects of ALA in human colonic epithelial cells NCM460 and human colorectal adenocarcinoma cells Caco-2 were studied. ALA significantly promoted NCM460 and Caco-2 migration, increased mucosal tight junction factors ZO-1 and OCLN expression, and ALA accelerated cell injury repair of both cells in wound healing assay. Western blot analysis indicated that ALA inhibited a variety of mitogen-activated protein kinase (MAPK) signaling pathways in the epithelial cells. In conclusion, ALA was beneficial to repair of intestinal epithelial injury by regulating MAPK signaling pathways.

## 1. Introduction

Intestinal epithelial cells form a selective barrier that separates luminal contents from underlying tissue. Wound healing of intestinal epithelial after injury is a dynamic biological process regulated by a complex network of microenvironments [1]. Studies have shown that a variety of cytokines (such as *α*-actinin and toll-like receptor [2]), regulatory peptides, and dietary factors [3] modulate intestinal epithelial wound healing. It is worth noting that redox balance is crucial for intestine homeostasis, and overproduction of ROS caused by oxidative upregulation or fluctuant mitochondrial function is related to intestinal epithelial injury [4].

*α*-Lipoic acid (ALA), a kind of natural dimercaptan antioxidant, is a compound commonly found in mitochondria, which plays an essential role in mitochondrial metabolism [5]. As a cofactor of enzymes, ALA is involved in glucose and lipid metabolism and regulates gene transcription [6]. As a metabolic antioxidant, ALA regulates NF-*κ*B signal transduction and protects against oxidative injury [7]. In addition, ALA can downregulate proinflammatory redox-sensitive signal transduction processes and has a certain anti-inflammatory effect [8]. Based on the above properties, ALA is applied in Alzheimer's disease, diabetic polyneuropathy, and obesity [9]. At present, the application of ALA in the intervention of intestinal damage is increasingly popular. The study of Guven et al. showed that ALA prevented ischemia/reperfusion injury in the rat intestine through scavenging ROS and RNS [10]. In an in vitro Caco-2 cell model, ALA supplementation was proved to enhance epithelial cell proliferation and thus prevented the disruption of intestinal epithelial integrity [11]. In an in vivo experiments on mice, ALA protected the intestine against ulcerative colitis and the associated systemic damage [12].

The present study aims to investigate the effect of ALA on intestinal epithelial injury repair and its mechanism, to provide theoretical basis for clinical treatment.

## 2. Materials and Methods

### 2.1. Cell Culture and Reagents

Human colonic epithelial cells NCM460 and human colorectal adenocarcinoma cells Caco-2 were purchased from the American Type Culture Collection (ATCC, Manassas, USA). The cells were cultured in Roswell Park Memorial Institute DMEM/F12 medium supplemented with 10% fetal bovine serum (FBS). Cells were maintained in a 37°C incubator with 5% CO_2_. All cell lines tested negative for mycoplasma. Alpha-lipoic acid (ALA) was purchased from Solarbio (Beijing, China). Based on the concentration range of ALA in other studies and the transport of ALA enantiomers in Caco-2 cells [13, 14], and combined with the results of pre-experiments, ALA was dissolved with DMSO and formulated to 50, 150 and 300 *μ*M for formal experiments.

### 2.2. Wound Healing Assay

Positioning marks were made with a permanent marker at the bottom of the cell culture plates to ensure that the same wound was observed. NCM460 and Caco-2 cells were cultured until 90% confluence in 6-well cell culture plates. Wounds were inflicted on the cell monolayers with 200-*μ*l pipette tips. Then cells were incubated with specific concentration of ALA in serum-free medium. During incubation, the cell migration was observed with an Olympus FluoView™ 300 confocal microscope (Tokyo, Japan). The wound areas were measured with ImageJ software. The remaining wound area was calculated using the following formula: (cell − free area at 12 h or 24 h/cell − free area at 0 h) × 100%. At least five fields were analyzed in each group.

### 2.3. Quantitative Real-Time PCR (qRT-PCR)

The total RNA was extracted from tissues using TRIzol Reagent (Thermo Fisher, USA) according to the manufacturer's instructions. Then RNA was reverse transcribed to cDNA with 1 *μ*g total RNA, using reverse transcriptase and Oligo dT primers (Takara, Japan). The cDNA was then amplified with specific primers by PCR. The conditions for qRT-PCR were as follows: 95°C for 3 min, followed by 40 cycles of 10 s at 95°C,10 s at 60°C, and 15 s at 70°C, followed by heating from 65°C to 95°C. Primers for qRT-PCR are listed as follows: ZO-1 forward primer 5′-GAA CGA GGC ATC ATC CCT AA-3′, reverse primer 5′-GAG CGG ACA AAT CCT CTC TG-3′. OCLN forward primer 5′-TTT GTG GGA CAA GGA ACA CA-3′, reverse primer 5′-TCA TTC ACT TTG CCA TTG GA-3′. GAPDH forward primer 5′-GAA GGT GAA GGT CGG AGT C-3′, reverse primer 5′-GAA GAT GGT GAT GGG ATT TC-3′.

### 2.4. Western Blot

Cells were washed with ice-cold PBS and lysed with RIPA buffer. Samples containing equal quantities of total proteins were resolved on 15% SDS polyacrylamide denaturing gel and transferred to nitrocellulose membranes. After blocking with 5% skim milk in TBST for 1 h, the membranes were incubated overnight at 4°C with the following primary antibodies: AKT (1 : 1000, Abcam, UK), JNK (1 : 1000, Abmart, Shanghai, China), and p38 (1 : 1000, Abmart, Shanghai, China). Goat anti-rabbit IgG secondary antibody (1 : 5000) (Servicebio, Wuhan, China) was used at room temperature for 1-h incubation. The blot was visualized by using ECL Chemiluminescence Kit (Epizyme, Shanghai, China). Each band was quantified via ImageJ software.

### 2.5. Statistical Analysis

The results of this study were expressed as mean ± SD values. Student's *t*-test was used to compare the results between the different groups. *P* < 0.05 was considered statistically significant. All analyses were performed using GraphPad Prism version 8 software.

## 3. Results

### 3.1. ALA Promoted Epithelial Cell Migration after Injury

**Figure 1:**
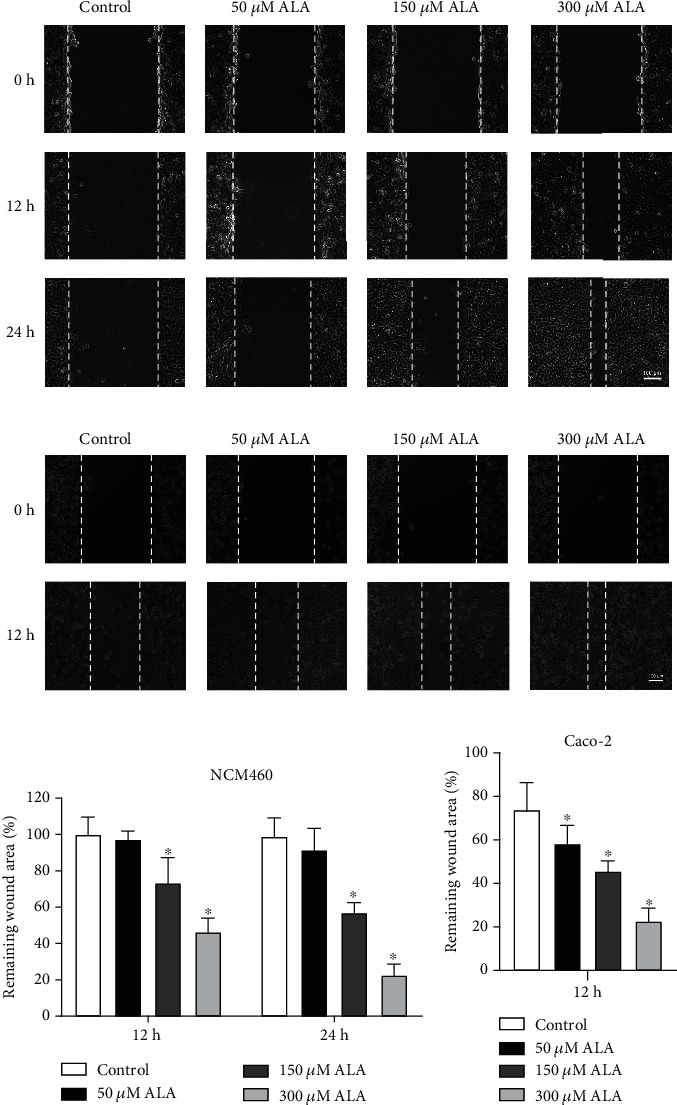
Restoration of epithelial cells treated with *α*-lipoic acid (ALA). (a) Representative images of cell wounds in wound healing assay of ALA (0–300 *μ*M) treated NCM460 cells. Wounds were created on the cell surface with the pipette tips, and then NCM460 cells were treated with ALA (0–300 *μ*M). The remaining wound areas were determined at 12 h and 24 h after wound generation. (b) Representative images of cell wounds in wound healing assay of ALA (0–300 *μ*M) treated Caco-2 cells. Wounds were created, and then Caco-2 cells were treated with ALA (0–300 *μ*M). The remaining wound areas were determined at 12 h after wound generation. (c, d) The remaining wound areas in NCM460 and Caco-2 cells treated with ALA (0-300 *μ*M). Data are presented as mean ± SD values of three duplicates. ∗*P* < 0.05.

Human colonic epithelial cells NCM460 and human colorectal adenocarcinoma cells Caco-2 were cultured in a serum-free medium for 12 h to form a monolayer. Compared with the control cells, the cells treated with ALA showed enhanced injury repair in a dose-dependent manner (Figures 1(a) and 1(b)). As shown in Figures 1(a) and 1(c), the remaining wound area of NCM460 cells that were treated with ALA (150 and 300 *μ*M) were significantly smaller than those of control cells after 12 h and 24 h of incubation (*P* < 0.05). Meanwhile, the remaining wound area of Caco-2 cells that were treated with ALA (50, 150, and 300 *μ*M) were significantly reduced after 12 h of incubation (Figures 1(b) and 1(d)) (*P* < 0.05).

### 3.2. ALA Increased the Expression of Tight Junction Factor in Intestinal Mucosa

**Figure 2:**
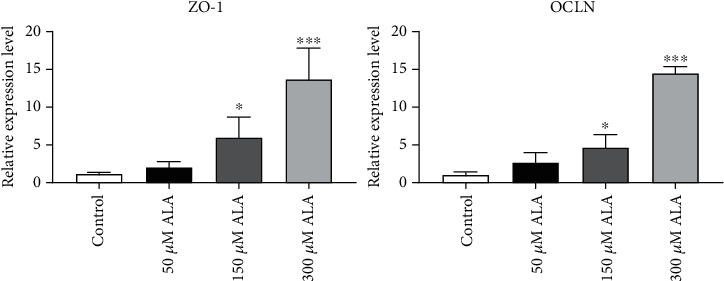
Effects of ALA on the mRNA expression of mucosal tight junction factors of NCM460 cells during injury repair. Quantitative real-time PCR analysis of tight junction protein 1 (ZO-1) and occludin (OCLN). ∗*P* < 0.05 and ∗∗∗*P* < 0.0001.

To reveal the effects of ALA on intestinal mucosal tight junction, the expression of ZO-1 and OCLN was measured at the mRNA level. As shown in Figure 2, in NCM460 cells incubated with ALA (150 and 300 *μ*M), the mRNA levels of both ZO-1 and OCLN were much higher than those in the control group (*P* < 0.05), whereas 50 *μ*M ALA had little influence on the expression of ZO-1 and OCLN (*P* > 0.05). ALA significantly increased the expression of ZO-1 and OCLN in the intestinal mucosa epithelial cells at the mRNA level.

### 3.3. ALA Promoted Intestinal Epithelial Injury Repair through Regulating PI3K/AKT, JNK, and p38 MAPK Signaling Pathways

**Figure 3:**
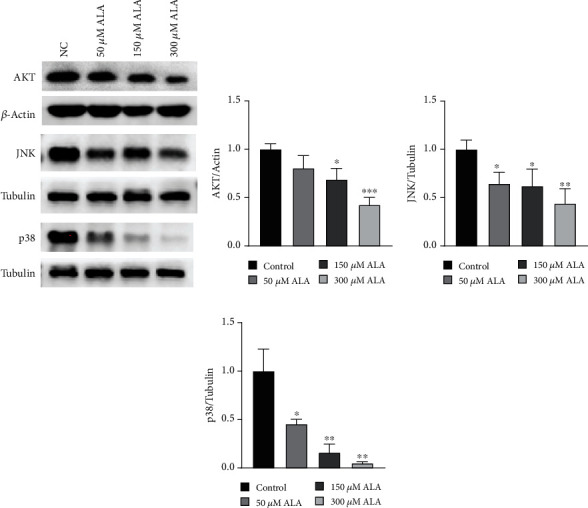
ALA suppressed the PI3K/AKT, JNK, and p38 MAPK signaling pathways in NCM460 cells. (a) Western blot showed that the activation of AKT, JNK, and p38 was suppressed by treatment with the ALA in NCM460 cells. (b) Quantified histograms of AKT protein levels normalized by actin. (c) Quantified histograms of JNK protein levels normalized by tubulin. (d) Quantified histograms of p38 protein levels normalized by tubulin. Data represent mean ± SD from at least three independent experiments. ∗*P* < 0.05, ∗∗*P* < 0.01, and ∗∗∗*P* < 0.001 compared to control.

Previous studies have shown that mitogen-activated protein kinases (MAPK)–related molecules are closely associated with wound healing in many cell lines, including epithelial cells, keratinocytes, and cancer cells [15–17]. To investigate the growth-promoting mechanism of ALA in epithelial cells, we assessed the status of MAPK signaling pathway in ALA-treated NCM460 cells. Western blot indicated that MAPK signaling-related molecules PI3K/AKT, Jun N-terminal kinases (JNK), and p38 were significantly suppressed by the treatment of ALA (Figure 3(a)). High concentrations of ALA suppressed AKT and JNK signaling pathways (Figures 3(b) and 3(c)), while the inactivation of p38 signaling pathway was observed at lower ALA concentrations (Figure 3(d)).

## 4. Discussion

Many factors such as inflammation, immunological factors, oxidative stress, medicines, and imbalance of gut microbiota may impair intestinal epithelium function and damage its barrier function [18, 19]. After intestinal epithelium injury, epithelial cells migrate, proliferate and differentiate, and heal gradually [20]. Clinical application of medicine to promote intestinal mucosal wound healing is beneficial to the rehabilitation of patients with various intestinal diseases, including inflammatory bowel diseases (IBD), celiac disease, and intestinal infections [21]. In the present study, we demonstrated that ALA could enhance intestinal injury repair and revealed its mechanism.

Lipoic acid, a powerful antioxidant existed in mitochondria, is absorbed through the gastrointestinal tract in vivo. Vegetables such as spinach, cauliflower, tomatoes, and carrots and meat such as liver are rich in lipoic acid, but food supplementation of lipoic acid is insufficient and slow to take effect. Therefore, ALA is commonly used as a drug or nutritional supplement for a variety of diseases [22].

Existing studies showed that ALA could accelerate mouse cutaneous wound healing [23] or promote human postoperative uterine healing [24]. However, its effect on intestinal epithelial wound healing remains unknown. In this study, we performed wound healing assay on both human colonic epithelial cells (NCM460) and colorectal adenocarcinoma cells (Caco-2). Low concentration of ALA (50 *μ*M) had no obvious effect on injury repair, while high concentration (150 and 300 *μ*M) of ALA could promote intestinal epithelial injury repair in a concentration-dependent manner (Figures 1(a) and 1(b)). In addition, ALA significantly enhanced epithelial cell migration (Figures 1(c) and 1(d)), which is an important process of wound repair.

Tight junction protein 1 (TJP1, also known as ZO-1), a membrane-associated cytoplasmic protein, plays an important role in cell-cell communication in the intercellular barrier in non-epithelial and epithelial cells [25]. ALA increased ZO-1 expression observed in our present study (Figure 2), which was closely related to proliferation and differentiation of epithelial cells. Occludin (OCLN) is another important tight junction protein in wound healing [26]. Studies have shown that increased OCLN can maintain intestinal barrier function in patients with ulcerative colitis and mice with colitis [27], which is consistent with our finding that ALA increased the expression of OCLN in intestinal epithelial injury repair (Figure 2).

As an antioxidant, the mechanism of ALA is related to its effect on oxidative stress. In a rat experiment, ALA pretreatment significantly reduced oxidative stress and inflammation in the intestine [28]. ALA protected piglet intestinal epithelium cells (IPEC-J2 cells) against H_2_O_2_ induced injury by scavenging hydroxyl radical [29]. However, the exact mechanism of ALA in intestinal epithelial injury repair process remains unclear.

Mitogen-activated protein kinases (MAPK)–related signaling pathways, including p38, JNK, ERK, and AKT, are involved in numerous cellular responses such us proliferation, differentiation, apoptosis, inflammation, and oxidative stress [30–32]. It has been found that MAPK-related signaling pathways were involved in wound healing. MAPK activation affected the proliferation, migration, and apoptosis of M1-like macrophages and delays the wound healing process after prostate surgery [33]. Glial cell line-derived neurotrophic factor (GDNF) promoted barrier maturation in immature enterocytes cells by inactivation of p38 MAPK signaling [34]. Delbue et al. found that inhibition of the ERK pathway rescued the tight junctional barrier defect in IEC cells [35]. Reactive oxygen species (ROS) is an important and common messenger produced in various environmental stresses and is known to activate many kinds of the MAPKs [36]. ALA may regulate MAPK signaling by eliminating excessive ROS produced after intestinal injury. In our study, we found that the repair of intestinal epithelial injury by ALA may be associated with the inactivation of the PI3K/AKT, JNK, and p38 MAPK signaling pathways (Figure 3), but further verification is needed. The difference in ALA inhibition of AKT, JNK, and p38 may be because they affect angiogenesis and inflammation in different pathways [37]. It has been reported that oxidative stress activates p38 MAPK signaling pathway and leads to a decrease in the expression of TJP [38]; this might be one of the reasons why ALA increased ZO-1 expression in our study.

## 5. Conclusion

In conclusion, we have demonstrated clearly that ALA increased NCM460 and Caco-2 cells proliferation and migration and increased the expression of tight junction factors ZO-1 and OCLN. Moreover, we found that the positive effect of ALA on intestinal epithelial injury repair may be related to MAPK signaling pathways. Our results provided a theoretical basis for the future development of ALA in treatment of intestinal injury.

## Data Availability

The datasets used and analyzed during the current study are available from the corresponding authors on reasonable request.

## References

[1] Iizuka M., Konno S. (2011). Wound healing of intestinal epithelial cells. *World Journal of Gastroenterology*.

[2] Harris G., Kuo Lee R., Chen W. (2006). Role of toll-like receptors in health and diseases of gastrointestinal tract. *World Journal of Gastroenterology*.

[3] Andou A., Hisamatsu T., Okamoto S. (2009). Dietary histidine ameliorates murine colitis by inhibition of proinflammatory cytokine production from macrophages. *Gastroenterology*.

[4] Aviello G., Knaus U. G. (2017). ROS in gastrointestinal inflammation: rescue or sabotage?. *British Journal of Pharmacology*.

[5] Packer L., Witt E. H., Tritschler H. J. (1995). Alpha-lipoic acid as a biological antioxidant. *Free Radical Biology & Medicine*.

[6] Solmonson A., DeBerardinis R. J. (2018). Lipoic acid metabolism and mitochondrial redox regulation. *The Journal of Biological Chemistry*.

[7] Packer L. (1998). *α*-Lipoic acid: a metabolic antioxidant which regulates NF-*κ*B signal transduction and protects against oxidative injury. *Drug Metabolism Reviews*.

[8] Maczurek A., Hager K., Kenklies M. (2008). Lipoic acid as an anti-inflammatory and neuroprotective treatment for Alzheimer’s disease. *Advanced Drug Delivery Reviews*.

[9] Tibullo D., Li Volti G., Giallongo C. (2017). Biochemical and clinical relevance of alpha lipoic acid: antioxidant and anti-inflammatory activity, molecular pathways and therapeutic potential. *Inflammation Research*.

[10] Guven A., Tunc T., Topal T. (2008). *α*-Lipoic acid and ebselen prevent ischemia/reperfusion injury in the rat intestine. *Surgery Today*.

[11] Varasteh S., Fink-Gremmels J., Garssen J., Braber S. (2018). *α*-Lipoic acid prevents the intestinal epithelial monolayer damage under heat stress conditions: model experiments in Caco-2 cells. *European Journal of Nutrition*.

[12] Trivedi P. P., Jena G. B. (2013). Role of *α*-lipoic acid in dextran sulfate sodium-induced ulcerative colitis in mice: studies on inflammation, oxidative stress, DNA damage and fibrosis. *Food and Chemical Toxicology*.

[13] Zhang W. J., Frei B. (2001). *α*‐Lipoic acid inhibits TNF‐a‐induced NF‐*κ*B activation and adhesion molecule expression in human aortic endothelial cells. *The FASEB Journal*.

[14] Uchida R., Okamoto H., Ikuta N. (2016). Investigation of enantioselective membrane permeability of *α*-lipoic acid in Caco-2 and MDCKII cell. *International Journal of Molecular Sciences*.

[15] Lee J., Jang H., Park S. (2019). Platelet-rich plasma activates AKT signaling to promote wound healing in a mouse model of radiation-induced skin injury. *Journal of Translational Medicine*.

[16] Saika S., Okada Y., Miyamoto T. (2004). Role of p38 MAP kinase in regulation of cell migration and proliferation in healing corneal epithelium. *Investigative Ophthalmology & Visual Science*.

[17] Harper E. G., Alvares S. M., Carter W. G. (2005). Wounding activates p38 map kinase and activation transcription factor 3 in leading keratinocytes. *Journal of Cell Science*.

[18] Bhattacharyya A., Chattopadhyay R., Mitra S., Crowe S. E. (2014). Oxidative stress: an essential factor in the pathogenesis of gastrointestinal mucosal diseases. *Physiological Reviews*.

[19] Weiss G. A., Hennet T. (2017). Mechanisms and consequences of intestinal dysbiosis. *Cellular and Molecular Life Sciences*.

[20] Dignass A. U. (2001). Mechanisms and modulation of intestinal epithelial repair. *Inflammatory Bowel Diseases*.

[21] Weichselbaum L., Klein O. D. (2018). The intestinal epithelial response to damage. *Science China. Life Sciences*.

[22] Salehi B., Berkay Yilmaz Y., Antika G. (2019). Insights on the use of alpha-lipoic acid for therapeutic purposes. *Biomolecules*.

[23] Leu J. G., Chen S. A., Chen H. M. (2012). The effects of gold nanoparticles in wound healing with antioxidant epigallocatechin gallate and *α*-lipoic acid. *Nanomedicine*.

[24] Sammour H., Elkholy A., Rasheedy R., Fadel E. (2019). The effect of alpha lipoic acid on uterine wound healing after primary cesarean section: a triple-blind placebo-controlled parallel-group randomized clinical trial. *Archives of Gynecology and Obstetrics*.

[25] Lee E.-Y., Yu J. Y., Paek A. R. (2020). Targeting TJP1 attenuates cell-cell aggregation and modulates chemosensitivity against doxorubicin in leiomyosarcoma. *Journal of Molecular Medicine (Berlin, Germany)*.

[26] Volksdorf T., Heilmann J., Eming S. A. (2017). Tight junction proteins claudin-1 and occludin are important for cutaneous wound healing. *The American Journal of Pathology*.

[27] Rawat M., Nighot M., Al-Sadi R. (2020). IL1B increases intestinal tight junction permeability by up-regulation of MIR200C-3p, which degrades occludin mRNA. *Gastroenterology*.

[28] Dadhania V. P., Tripathi D. N., Vikram A., Ramarao P., Jena G. B. (2010). Intervention of *α*-lipoic acid ameliorates methotrexate-induced oxidative stress and genotoxicity: a study in rat intestine. *Chemico-Biological Interactions*.

[29] Cai X., Chen X., Wang X. (2013). Pre-protective effect of lipoic acid on injury induced by H2O2 in IPEC-J2 cells. *Molecular and Cellular Biochemistry*.

[30] Cargnello M., Roux P. P. (2011). Activation and function of the MAPKs and their substrates, the MAPK-activated protein kinases. *Microbiology and Molecular Biology Reviews*.

[31] Yue J., Lopez J. M. (2020). Understanding MAPK signaling pathways in apoptosis. *International Journal of Molecular Sciences*.

[32] Wagner E. F., Nebreda A. R. (2009). Signal integration by JNK and p38 MAPK pathways in cancer development. *Nature Reviews. Cancer*.

[33] Deng Z., Shi F., Zhou Z. (2019). M1 macrophage mediated increased reactive oxygen species (ROS) influence wound healing via the MAPK signaling in vitro and in vivo. *Toxicology and Applied Pharmacology*.

[34] Meir M., Flemming S., Burkard N. (2015). Glial cell line-derived neurotrophic factor promotes barrier maturation and wound healing in intestinal epithelial cells in vitro. *American Journal of Physiology. Gastrointestinal and Liver Physiology*.

[35] Delbue D., Lebenheim L., Cardoso-Silva D. (2021). Reprogramming intestinal epithelial cell polarity by Interleukin-22. *Frontiers In Medicine*.

[36] Jalmi S. K., Sinha A. K. (2015). ROS mediated MAPK signaling in abiotic and biotic stress- striking similarities and differences. *Frontiers in Plant Science*.

[37] Yu G., Yu H., Yang Q. (2022). Vibrio harveyi infections induce production of proinflammatory cytokines in murine peritoneal macrophages via activation of p 38 MAPK and NF-*κ*B pathways, but reversed by PI3K/AKT pathways. *Developmental and Comparative Immunology*.

[38] Yu J., Liu F., Yin P. (2013). Involvement of oxidative stress and mitogen-activated protein kinase signaling pathways in heat stress-induced injury in the rat small intestine. *Stress*.

